# NVVC/NHJ Durrer prizes 2016

**DOI:** 10.1007/s12471-017-0975-2

**Published:** 2017-02-28

**Authors:** J. J. Piek

**Affiliations:** Department of Cardiology, Academic Medical Centre, Amsterdam, The Netherlands

The NVVC/NHJ publication prizes are named after one of founding fathers of Dutch cardiology, Professor D. Durrer (1918–1984), who was the chair of the department of Cardiology at the former Wilhelmina Gasthuis in Amsterdam. He was an outstanding pioneer in the field of electrophysiology with classical work on the electric activation of the heart in the 1960s and 1970s [1]. He was also the founder of the Interuniversity Cardiology Institute of the Netherlands (ICIN) in 1972. Moreover, the Durrer Center, founded in 2008, carries his name in his memory. The Durrer Center is a national multidisciplinary collaboration of academic research institutes in the field of cardiology, genetics and biostatistics.

The Netherlands Heart Journal, the official journal of the Netherlands Society of Cardiology (NVVC), awarded the Durrer prizes to two outstanding NHJ articles published in 2016. These articles are selected based upon their originality and scientific quality as well as the number of citations. One article is basically oriented and the other article has a clinical focus. These two articles were selected from a to﻿tal of 108 articles published in NHJ in 2016.

The best basically oriented article was entitled ‘Animal model of heart failure with preserved ejection fraction’ [2]. It is a review of the available experimental models varying from rodents to large animals to study the effect of heart failure with a preserved ejection fraction (HFpEF). HFpEF is a clinical syndrome of heart failure characterised by a preserved systolic function of the left ventricle. Signs of heart failure are determined by diastolic dysfunction and it is the underlying mechanism of almost 50% of the patients with heart failure. The pathophysiology of HFpEF is poorly understood because of the limited access to hu﻿man material using myocardial biopsies. Moreover, there is a lack of animal models that mimic the human pathology, while these experimental models are valuable to clarify subcellular and molecular mechanisms determining HFpEF. A better insight into the mechanism of HFpEF is of crucial importance for instalment of effective therapy. This awarded review summarises the advantages and disadvantages of the available experimental models.

The best clinical article is entitled ‘Patients with heart failure with preserved ejection fraction and low levels of natriuretic peptides’ [3]. This clinical study is a nice illustration of the problems encountered in the management of patients with signs of heart failure and HFpEF. This study evaluates the levels of beta-type natriuretic peptides (BNP) that are used for the diagnosis of heart failure. A total of 157 patients were enrolled in the Coordinating study evaluating Outcomes of Advising and counselling in Heart failure (COACH) with a preserved left ventricle ejection fraction (LVEF ≥40%). A total of 30 patients had a BNP <100 pg/ml and were compared with 127 patients with an elevated BNP ≥100 pg/ml. All the analyses were repeated for NT-proBNP (<300 pg/ml and ≥300 pg/ml) to validate the findings of the BNP levels. The results show that characteristics such as age, sex and LVEF, biomarkers and comorbidities did not differ between the patients with a low BNP level compared with a high BNP level. Patients with a low BNP level had a higher body mass index and lower cardiac troponin I level, while these patients used less diuretics and beta-blockers. There were no differences with respect to quality of life and heart failure related symptoms. The authors conclude that patients with clinically diagnosed HFpEF and a low BNP are similar to those patients with an elevated BNP level with the main exception of a higher body mass index.

The first authors of these articles received an educational grant, provided by the NVVC, at the Annual Spring Meeting of the NVVC held at the congress centre the Leeuwenhorst at Noordwijkerhout on 6 and 7 April 2017. The NHJ would like to congratulate the authors with these awards and thank them for submitting their excellent work to our journal. The editorial board of the NHJ hopes that the Durrer prizes are a stimulus for authors to send their best papers to our journal.

## References

[1] Durrer D, van Dam RT, Freud GE, Janse MJ, Meijler FL, Arzbaecher RC (1970). Total excitation of the isolated human heart. Circulation.

[2] Conceição G, Heinonen I, Laurenço AP, Duncker DJ, Falcão-Pires I (2016). Animal model of heart failure with preserved ejection fraction. Neth Heart J.

[3] Meijers WC, Hoekstra T, Jaarsma T, van Veldhuisen DJ, de Boer RA (2016). Patients with heart failure with preserved ejection fraction and low levels of natriuretic peptides. Neth Heart J.

